# Stability of Circulating Blood-Based MicroRNAs – Pre-Analytic Methodological Considerations

**DOI:** 10.1371/journal.pone.0167969

**Published:** 2017-02-02

**Authors:** Charlotte Glinge, Sebastian Clauss, Kim Boddum, Reza Jabbari, Javad Jabbari, Bjarke Risgaard, Philipp Tomsits, Bianca Hildebrand, Stefan Kääb, Reza Wakili, Thomas Jespersen, Jacob Tfelt-Hansen

**Affiliations:** 1 Department of Cardiology, the Heart Centre, Copenhagen University Hospital, Rigshospitalet, Copenhagen, Denmark; 2 Department of Medicine I, University Hospital Munich, Campus Grosshadern, Ludwig-Maximilians University Munich (LMU), Munich, Germany; 3 Cardiovascular Research Center, Massachusetts General Hospital, Boston, MA, United States of America; 4 DZHK (German Centre for Cardiovascular Research), Partner Site Munich, Munich Heart Alliance (MHA), Munich, Germany; 5 Department of Biomedical Sciences, University of Copenhagen, Copenhagen, Denmark; University of Texas MD Anderson Cancer Center, UNITED STATES

## Abstract

**Background and aim:**

The potential of microRNAs (miRNA) as non-invasive diagnostic, prognostic, and predictive biomarkers, as well as therapeutic targets, has recently been recognized. Previous studies have highlighted the importance of consistency in the methodology used, but to our knowledge, no study has described the methodology of sample preparation and storage systematically with respect to miRNAs as blood biomarkers. The aim of this study was to investigate the stability of miRNAs in blood under various relevant clinical and research conditions: different collection tubes, storage at different temperatures, physical disturbance, as well as serial freeze-thaw cycles.

**Methods:**

Blood samples were collected from 12 healthy donors into different collection tubes containing anticoagulants, including EDTA, citrate and lithium-heparin, as well as into serum collection tubes. MiRNA stability was evaluated by measuring expression changes of miR-1, miR-21 and miR-29b at different conditions: varying processing time of whole blood (up to 72 hours (h)), long-term storage (9 months at -80°C), physical disturbance (1 and 8 h), as well as in a series of freeze/thaw cycles (1 and 4 times).

**Results:**

Different collection tubes revealed comparable concentrations of miR-1, miR-21 and miR-29b. Tubes with lithium-heparin were found unsuitable for miRNA quantification. MiRNA levels were stable for at least 24 h at room temperature in whole blood, while separated fractions did show alterations within 24 h. There were significant changes in the miR-21 and miR-29b levels after 72 h incubation of whole blood at room temperature (p<0.01 for both). Both miR-1 and miR-21 showed decreased levels after physical disturbance for 8 h in separated plasma and miR-1 in serum whole blood, while after 1 h of disturbance no changes were observed. Storage of samples at -80°C extended the miRNA stability remarkably, however, miRNA levels in long-term stored (9 months) whole blood samples were significantly changed, which is in contrast to the plasma samples, where miR-21 or miR-29b levels were found to be stable. Repetitive (n = 4) freeze-thaw cycles resulted in a significant reduction of miRNA concentration both in plasma and serum samples.

**Conclusion:**

This study highlights the importance of proper and systematic sample collection and preparation when measuring circulating miRNAs, e.g., in context of clinical trials. We demonstrated that the type of collection tubes, preparation, handling and storage of samples should be standardized to avoid confounding variables influencing the results.

## Introduction

The potential importance of circulating microRNA (miRNA) as non-invasive diagnostic, prognostic, and predictive biomarkers, as well as therapeutic targets has been given great attention [1–7]. MiRNAs are a class of small non-protein-coding RNA molecules that play an essential role in gene regulation at the post-transcriptional level by causing messenger RNA (mRNA) degradation or inhibiting mRNA translation into protein [8–13]. More than 2,500 miRNAs have been found in humans [14], and these appear to regulate 60% of the human protein-coding genes [15]. With the discovery of miRNAs being expressed in clinical samples such as plasma/serum [2,16–20], urine [21,22], and other bodyfluids [23,24] miRNAs have evolved as potential biomarkers that may provide insights into the underlying pathophysiological condition of a disease. Furthermore miRNAs have the potential of being used for diagnostic purposes, and thereby guiding treatment strategies. However, when used as clinical biomarkers, attention must be paid to the stability of miRNAs given the various conditions the clinical samples are processed with respect to the specific methodology and source of biological material (e.g., whole blood, plasma or serum) [25,26]. While RNA molecules are known to be highly unstable, previous studies have indicated that miRNAs are remarkably stable in plasma and serum, and resistant to RNase activity, as well as extreme pH and multiple numbers of freeze-thaw cycles [2,16,17]. However, studies characterizing and quantifying biomarkers in heart failure and atrial fibrillation patients were lacking consistent and reproducible results concerning quantification of the miRNA concentration in blood samples of patients [27]. One possible explanation could be an inconsistency in the methodology used. Previous studies have highlighted that confounding methodological factors should be ruled out [25,26,28]. To our knowledge, no study has described the methodology of sample preparation and storage systematically with respect to miRNAs as blood biomarkers. It is not known whether the time between blood collection and processing of plasma/serum or physical disturbance of the samples affects the miRNA levels and the reproducibility of results. Therefore, the aim of this study was to investigate the stability of miRNA expression in whole blood and plasma/serum under various relevant clinical and research conditions in several types of collection tubes.

## Materials and Methods

### Study population and blood sample collection

This study was conducted in two different centers (Copenhagen, Denmark and Munich, Germany). Blood samples were collected from 6 healthy donors (4 males and 2 females) in Copenhagen, and 6 healthy donors (3 males and 3 females) in Munich. All were of Caucasian ethnicity, except of 2 Asians in the Copenhagen cohort. For each study, whole blood was collected by venous puncture with butterfly needles into 6 ml serum separator and/or anticoagulant (salts of EDTA, lithium-heparin (at both study sites) and citrate (only at Copenhagen site)) containing tubes at the same blood draw. All tubes were inverted 10 times for mixing immediately after collection, and before further processing and storage. Participants provided written informed consent before participation, and the study was conducted according to the guidelines of The National Committee on Health Research Ethics, Denmark, and The Local Ethics Committee of the University of Munich, Germany. All data used in this study were analyzed anonymously and according to the health research ethics committee system in Denmark and the project did not need approval by the committees. Samples in Munich were collected in a general biobanking effort that was approved by the local ethics committee.

### Sample preparation and storage

To obtain plasma and serum, whole blood samples were either centrifuged at 3,000 revolution per minute (rpm) at room temperature for 15 minutes (min) in Copenhagen or at 4,000 rpm for 20 min at room temperature in Munich. The supernatant was immediately and carefully removed with 1 cm from the buffy coat, and frozen in 1.8 ml RNase-free cryo-tubes, and stored at -80°C until further processing. Plasma was obtained as the cell-free supernatant remaining after centrifuging blood, collected in the presence of an anticoagulant. Serum was collected as the cell-free supernatant, accessible after centrifuging blood incubated at room temperature for at least 30 min between collection and centrifugation to allow the blood in the serum separator tubes to clot spontaneously.

### RNA preparation

Total RNA was purified from a 300 μl sample, and the extraction was followed according to the NucleoSpin® (Macherey-Nagel, Germany) miRNA plasma protocol [29]. *C*. *elegans* miR-39 (cel-miR-39) was spiked-in as a control for extraction efficiency, as previous described [16]. All samples were eluted in 60 μl of RNase/DNase free water and stored at -80°C until further analysis.

### Reverse transcriptase reactions

Since the expression of miRNAs in the blood is very low, spectrophotometry is not reliable for quantification. In order to compensate for potential variations we added cel-miR-39 as a loading control (see above) and used a fixed volume of input RNA eluate as it was described before [16]. Total cDNA was synthesized from total RNA (5 μl), following the manufacturer´s instructions. TaqMan MicroRNA Reverse Transcription Kit (Life Technologies, USA) was used for multiplex reverse transcription reactions carried out with the primer sets for hsa-miR-1 (Life Technologies, USA), hsa-miR-21 (Life Technologies, USA), hsa-miR-29b (Life Technologies, USA) and cel-miR-39 (Life Technologies, USA) (used as loading control for extraction efficiency). The reactions were incubated in a Research Thermal Cycler (Applied Biosystems, USA (at Copenhagen site) or Bio Rad iQ, Germany (at Munich site)) for 30 min at 16°C, 30 min at 42°C, 5 min at 85°C and then held at 4°C.

### Quantitative real-time polymerase chain reaction (qRT-PCR)

qRT-PCR was perfomed using TaqMan Gene Expression MasterMix (Life Technologies, USA) and TaqMan probes (Life Technologies, USA) for hsa-miR-1 (Assay-ID: 000385), hsa-miR-21 (Assay-ID:000397), hsa-miR-29b (Assay-ID: 000413) and cel-miR-39 (Assay-ID: 000200) according to manufacturer’s instructions under sterile conditions using either an Applied Biosystems 7300 Real-Time PCR System (Copenhagen samples) or a CFX 96 C1000 touch Realtime Cycler (Bio Rad, Germany) (Munich samples). Reactions were run as triplicates using a total reaction volume of 10 μl. Thermal cycling was performed as follows: 1. 95°C for 10 minutes, 2. 95°C for 15 seconds, 3. 60°C for 60 seconds. Step 2 and 3 were repeated 44 times. The quantitative endpoint of the qRT-PCR was the threshold cycle (C_T_), defined as the PCR cycle at which the fluorescent signal of the PCR product crosses the detection threshold (set at 0.2 units).

### Experimental procedures

#### Different blood tubes

Blood was directly collected into different blood tubes containing serum separator and/or anticoagulant (salts of EDTA, lithium-heparin (at both study sites) and citrate (only at Copenhagen site)). Blood was processed to plasma/serum and RNA was isolated.

#### Impact of delayed processing

Whole blood samples were collected as described above and were stored at room temperature up to 72 hours (h) and then used for RNA isolation. Plasma and serum samples were processed immediately after blood collection and separated fractions were incubated for 24 h or 4 days (d) before RNA was isolated.

#### Impact of disturbance

Blood samples were placed on a PMR 30 Mini Rocker Shaker (Grant Instruments, UK). After 1 and 8 h of shaking at 30 rpm RNA was isolated.

#### Long-term storage

Samples of whole blood from the same blood draw, collected in EDTA tubes, were either processed to plasma or left untouched, and the samples were stored in parallel at -80°C for either 1 day or 9 months prior to analysis.

#### Repetitive freeze-thaw cycles

Blood samples were processed immediately after blood collection and aliquots of plasma and serum were stored at -80°C. One aliquot was thawed once, another aliquot was thawed and frozen four times before RNA was isolated.

#### Blood fractions

Samples of EDTA-treated whole blood from 3 subjects were separated into three fractions: plasma, buffy coat, and red blood cells (RBC). The levels of miR-21 and miR-29b were quantified in each of these fractions

### Data analysis

The qRT-PCR data, miRNA quantification C_T_, were collected by SDS1.2 software (Applied Biosystems, USA) and imported into Microsoft Excel, and the mean CT-values were calculated. Normalized ΔC_T_-values were calculated by subtracting the mean C_T_-value of cel-miR-39 from the mean C_T_ value of the target miRNA for each sample.

### Statistical analysis

**Fig 1:**
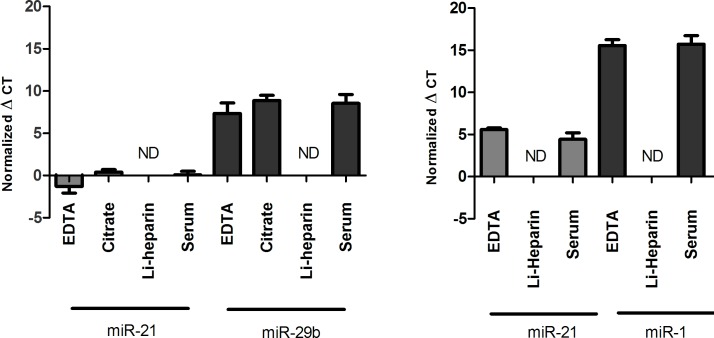
Different blood tubes for miRNA analysis. miRNA was isolated and levels of miR-1, miR-21 and miR-29b from either; EDTA-plasma, citrate-plasma, lithium-heparin-plasma, or serum fraction, were detected by RT-qPCR. Values were normalized to a spike-in control, cel-miR-39. Normalized average ΔC_T_ values for each are shown, demonstrating amplification from all sources, except lithium-heparin-plasma. In the remaining samples, the levels of miR-21 and miR-29b did not vary significantly between the blood tubes. Li = lithium, ND = not detectable. A) Copenhagen Cohort. B) Munich Cohort.

**Fig 2:**
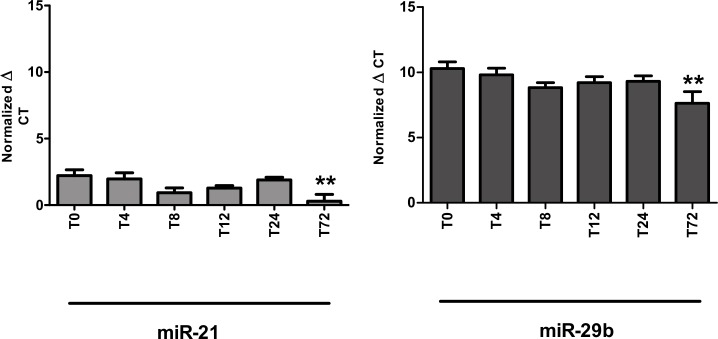
Stability of miRNA in whole blood incubated at room temperature. Whole blood was collected into EDTA containing tubes and incubated for 0, 4, 8, 12, 24 and 72 h at room temperature before processed into plasma. qRT-PCR was performed. Statistically significant differences in miRNA expression are marked with asterisks (**). **p<0.01. A) miR-21. B) miR-29b.

**Fig 3:**
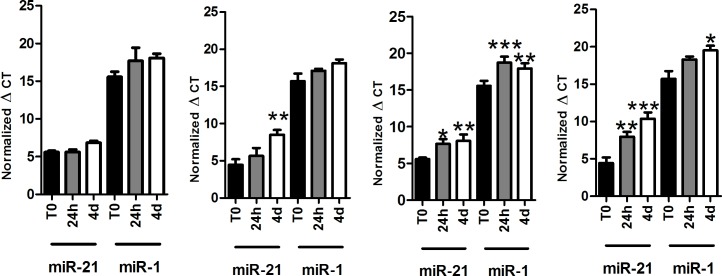
Impact of delayed processing on separated blood fractions. Whole blood was collected into EDTA or serum separator containing tubes and incubated at room temperature for 0h, 24h, and 4 days before processed into plasma (A) and serum (B) or processed into plasma (C) and serum (D) immediately and then incubated at room temperature for 0h, 24h, and 4d. Data presented as normalized average ΔC_T_values±SEM. *p<0.05, **p<0.01, ***p<0.001.

**Fig 4:**
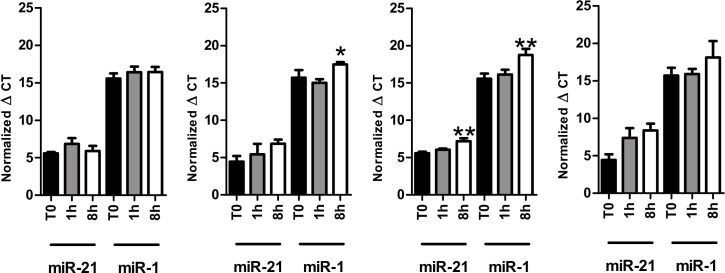
Impact of physical disturbance. Whole blood was collected into EDTA or serum separator containing tubes and incubated on a shaker at 30 rpm at room temperature for 0h, 1h, and 8h before processed into plasma (A) and serum (B) or processed into plasma (C) and serum (D) immediately and then incubated on a shaker at 30 rpm at room temperature for 0 h, 1 h, and 8 h. Data presented as normalized average ΔC_T_values±SEM. *p<0.05, **p<0.01, One-way ANOVA with Dunnet post test.

**Fig 5:**
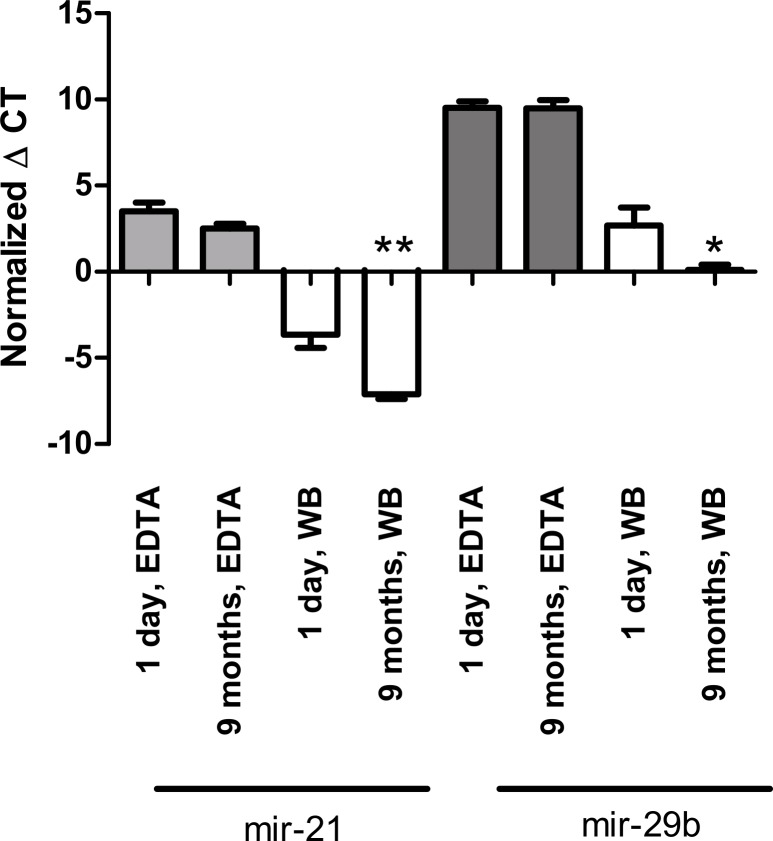
Incubation of plasma at -80°C for up to 9 months. There was no significant change in miRNA concentration up to 9 months after freezing for either miR-21 (A) or miR-29b (B) in plasma (p = 0.11 and p = 0.96). However, the concentration of both miRNAs in whole blood increased during storage at -80°C. *p<0.05, **p<0.01.

**Fig 6:**
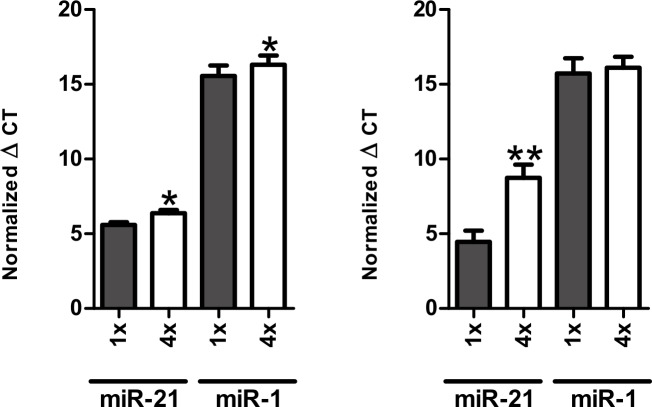
MiRNA stability after repetitive freeze-thaw cycles within the separated fractions. Whole blood was collected into EDTA or serum separator containing tubes, immediately processed into plasma (A) and serum (B) and frozen at -80°C. Samples were thawed one or four times before RNA was isolated and RT-qPCR was performed. *p<0.05, **p<0.01, unpaired student t-test.

**Fig 7:**
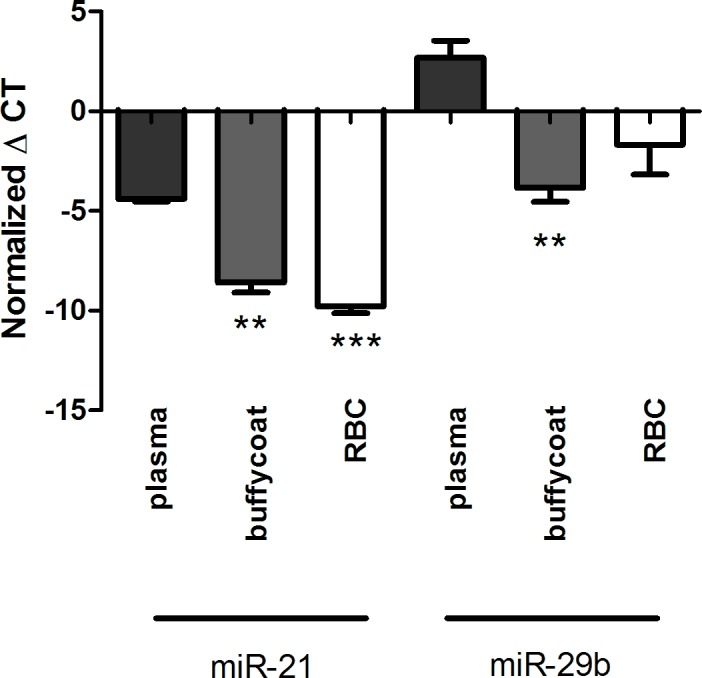
MiRNA levels in the 3 blood fractions; plasma, buffy coat and red blood cells (RBC). The two miRNAs, miR-21 and miR-29b, were differently distributed in the fractions; there are significant higher levels of miR-21 in the buffy coat and RBC compared to plasma, and miR-29b plasma levels are significantly lower than in the buffy coat. Statistically significant differences in miRNA expression are marked with asterisks (*).**p<0.01, ***p<0.001.

Statistical analysis was performed using one-way analysis of variance (ANOVA), repeated measures ANOVA, and where indicated post-hoc pairwise comparisons with Dunnett´s test, and paired Student´s t-test. One-way ANOVA was applied to compare the miRNA levels between blood tubes (Fig 1A and 1B) and storage at room temperature was assessed by the repeated measures ANOVA test (Fig 2A and 2B). Impact of delayed processing and physical disturbance (Fig 3A–3D), and blood fractions (Fig 4A–4D) were measured by one-way ANOVA. Paired two-tailed t-test was applied to compare the miRNA levels between different samples (Figs 5, 6A, 6B and 7). A two sided P-value of <0.05 was considered statistically significant. Analyses were performed with SAS version 9.4 (SAS institute, USA) and GraphPad PRISM 5.0 (GraphPad Software, USA).

## Results

### Different types of blood tubes for microRNA analysis

The following analysis describes the impact of the distinct blood preparation procedures with respect to tube types used in this study. At both sites blood from 9 subjects was drawn into different vacutainer tubes containing either serum separator or one of the following anticoagulants: EDTA, lithium-heparin, or citrate (Copenhagen site only). MiR-1, miR-21 and miR-29b could be detected in blood from all sources, except from lithium-heparin plasma (Fig 1). In the remaining samples, the levels of miR-1, miR-21, and miR-29b did not vary significantly between the blood tubes (p>0.05 for all).

### Incubation of whole blood at room temperature for extended periods of time

To mimic clinical blood sample handling, the stability of miRNAs incubated at room temperature for various duration was evaluated. In the Copenhagen cohort we measured miRNA levels in EDTA-treated whole blood samples. At different experimental time points (0, 4, 8, 12, 24 and 72 h) whole blood was processed to plasma (Fig 2). These time points were chosen to represent typical short-term transport and storage conditions in the clinical laboratory. Significant changes in the miRNA levels after 72 h were observed for miR-21 (p<0.01) and miR-29b (p<0.01). However, miRNA concentrations were stable in whole blood at room temperature for at least 24 h (p = 0.50 for miR-21 and p = 0.16 for miR-29b compared to T0).

In the Munich cohort we evaluated time-dependent effects on miR-1 and miR-21 levels in already separated fractions of EDTA plasma and serum plasma samples in addition to whole blood samples at room temperature after 24 h and 4 d (Fig 3). We could confirm the results from the Copenhagen cohort demonstrating that miR-21 levels in EDTA-treated whole blood are stable for at least 24 h (Fig 3A). In tubes containing serum separator miR-21 was stable only for up to 24 h (Fig 3B) whereas miR-1 levels were stable up to 4 d. Separated plasma or serum fractions showed significantly reduced miRNA levels after incubation for 24 h or 4 d (Fig 3C and 3D).

### MiRNA stability during physical and mechanical disturbance

To evaluate the effect of physical disturbances (e.g. transportation of blood samples from one center to another center) different samples (EDTA whole blood, serum whole blood, EDTA plasma fraction, serum fraction) were placed on a shaker for 1 or 8 h (Fig 4). The miRNA expression of both miR-1 and miR-21 decreased significantly after 8 h of physical disturbance in the separated plasma fraction (Fig 4C). In the serum fraction the results showed a more stable profile revealing just a slight non-significant trend for a decreased concentration over time (Fig 4D). Furthermore, we compared if the effect of physical disturbance differs in whole blood vs. the separated fractions (plasma and serum). MiRNA expression in EDTA-treated whole blood was stable after 1 or 8 h of disturbance (Fig 4A), in serum whole blood we observed a significant reduction in miR-1 expression after 8 h whereas miR-21 expression remained stable (Fig 4B). These results suggest no preventive effect of fraction separation prior to mechanical stress.

### Storage of plasma vs. whole blood at -80°C

Next, we investigated whether long term storage at -80°C had any effect on miRNA stability, by examining the effect of storage conditions on miRNA expression. The analysis revealed that storage of separated plasma at -80°C for 9 months had no significant effect on miR-21 and miR-29b levels (p = 0.11 and p = 0.96, respectively), while the concentration of free miRNAs in whole blood increased during storage at -80°C for both miRNAs over time (+3.47±0.83, p<0.01 for miR-21 and +2.56±1.09, p = 0.04 for miR-29b) (Fig 5).

### MiRNA stability after repetitive freeze-thaw cycles within the separated fractions

In order to determine the reproducibility of results in laboratory routine and to evaluate the stability of miRNAs in plasma and serum fractions with respect to repetitive freeze-thaw action we performed another series of experiments. Using a sample that was stored at -80°C and thawed once as a reference a decrease in miRNA-concentrations after 4 cycles of freeze-thaw was observed (Fig 6). These concentration changes were present for miR-21 (5.59±0.19 vs. 6.38±0.21, p<0.05) and miR-1 (15.57±0.69 vs. 16.31±0.62, p<0.05) in EDTA plasma samples as well as for miR-21 (4.45±0.75 vs. 8.74±0.88, p<0.01) in serum samples.

### MiRNA levels in fractionated blood

The levels of miR-21 and miR-29b were quantified in plasma, buffy coat, and RBC. We found that the two miRNAs were differently distributed in the 3 fractions (Fig 7). We observed significantly higher levels of miR-21 in the buffy coat (+4.20±0.51, p<0.01) and RBC (+5.39±0.37, p<0.001) compared to plasma, and miR-29b plasma levels are significantly lower than in the buffy coat samples (+6.54±1.10, p<0.01).

## Discussion

This study highlights several variables that could affect the concentration of miRNAs in whole blood and in plasma/serum samples, respectively. To our knowledge, this study is the first to provide detailed methodological information regarding the role of miRNAs as biomarkers, which have to be taken into account for generating reliable and representative data for use in clinical routine. We demonstrated that the type of collection tubes, preparation, handling and storage of samples should be standardized to avoid confounding variables influencing the results and to provide a solid basis for comparison of results between studies.

### Blood collection and preparation

Collection of whole blood is the first step in the preparation of plasma and serum, and is important for accurate analysis of miRNA expressions. The blood tubes containing serum separator or the anticoagulants, EDTA and citrate, do not significantly change the detectible levels of miRNAs. In contrast, the presence of lithium-heparin makes detection of the three miRNAs impossible. Both plasma and serum samples have been used for circulating miRNA analysis [20], and although all tubes used in our study were effective (except lithium-heparin), there were subtle differences for miRNA-expression in EDTA plasma vs. citrate plasma vs. serum fraction samples. Previous studies have shown that different centrifugation protocols can produce platelet-rich or platelet-poor plasma, which could lead to different amounts of blood cell contamination in these fluids [30], and the coagulation process may also affect the concentration of miRNAs [26]. Furthermore, the samples of the Munich cohort showed a larger variability of serum results in comparison to plasma EDTA (data not shown). This data indicates that samples collected from different blood tubes and blood fractions should not be included in the same study or directly compared to each other, in order to avoid confounding of the results.

### Stability of miRNA in whole blood before processing to plasma

Previous studies have shown that plasma miRNAs are stable under various conditions [16,20]. We found a significant change in the miR-21 and miR-29b levels after 72 h of incubation of whole blood at room temperature, yet no changes were observed after 24 h at room temperature. These findings indicate that miRNAs may be released from blood cells and platelets, and argues for storing only processed samples (plasma or serum) rather than whole blood. Our study is in line with previous studies reporting only small variations in plasma miRNA levels at room temperature up to 24 h [2,20], and indicates that the storage of whole blood at room temperature under clinical conditions for extended periods of time (at least up to 24 h) prior to the separation of plasma does not appear to affect plasma miRNA concentrations. Nevertheless, separated fractions of plasma seem to be less stable showing significant changes already within 24 h after blood collection and separation. In view of these results it is important to keep in mind that samples should still be processed as fast as possible or in a systemic temporal fashion. In case of a longer blood processing periods whole blood samples should be preferred. Still, the possibility that the stability of distinct miRNAs may be different should be taken into account; the result of this study only reflects the stability of miR-1, miR-21, and miR-29b.

### MiRNA stability during physical disturbance

Physical disturbance resulted in decreased levels of miRNAs in whole blood and separated fractions of plasma and serum. The difference was only significant for serum whole blood and separated plasma after 8 h of disturbance, but a trend towards a decrease over time was detectable for all the measurements. However, whole blood samples seem to be more robust than the separated fractions in conditions of physical stress, but they still show a similar trend of decreasing miRNA levels over time. So far, no report on miRNA stability in whole blood, plasma or serum during transport or physical disturbance has been published. The reason for the reduced miRNA expression after disturbance remains highly speculative. It is not very likely that contaminating cells (e.g. platelets) are the reason since lysis of cells would result in increased miRNA expression. A potential explanation is plasma inhibitors that might be activated by mechanical disturbance, i.e. shaking might provide sufficient activation energy for inhibitors to become active. Nevertheless, translating our results into a clinical practice long-term physical disturbance of blood samples, e.g., in context of transportation, should be avoided to prevent artificial altered results of miRNA concentration.

### Storage at -80°C

In our study long-time storage at -80°C had no significant effect on miRNA expression up to 9 months after freezing for either miR-21 or miR-29b in EDTA plasma samples. In contrast, increased levels of free miRNA in whole blood samples were found 9 months after freezing. These findings indicate that miRNAs may be released from blood cells and platelets, and argues for storing only processed samples (plasma or serum) rather than whole blood. Additionally, a previous study has shown that hemolysis during sample preparation can change the level of miRNAs, producing artificially high miRNA concentrations [31].

### Repetitive freeze-thaw cycles

MiRNA levels (miR-1 and miR-21) were decreased after four repetitive freeze-thaw cycles in plasma and serum. A similar effect has been shown previously by Zhao et al. [32]. They evaluated miR-346 and miR-134 and could demonstrate reduced miRNA levels in samples that were frozen/thawed. The effect observed in our study was not as striking but may be mimic a biological signal. Interestingly, Farina et al. observed the opposite effect [33]. In their study the serum expression of several miRNAs, including miR-21, was increased after two freeze-thaw cycles. These results suggest that multiple measurements of a frozen sample should be done with caution for direct comparison of results. Assuming a systematic effect, the relative changes between groups should be less pronounced, but are not predictable.

### MiRNA levels in the three separated blood fractions

Human blood consists of RBC (erythrocytes), platelets, and white blood cells (leukocytes, buffy coat) suspended in plasma. We found that both miR-21 and miR-29b are present in all 3 blood fractions and that they are differentially distributed in the fractions. This underscores the danger of contamination of the plasma by cells from the cellular pellet during aspiration or hemolysis.

### Limitations

In the current study we focused on miR-1, miR-21 and miR-29b, however, we do acknowledge that the stability of these miRNAs may not be representative of all miRNAs in plasma, including miRNAs with either higher or lower expression level. Whether these results can be extrapolated to other miRNAs requires additional studies. Furthermore, we only studied a small number of individuals, which bears the potential of false-negative results due to insufficient statistical power. However, even within this small number of individuals we were able to identify potential variables affecting results which should be considered before initiating a clinical study. The majority of the study persons were Caucasians. Therefore, our results cannot be generalized to other non-Caucasian individuals. It could be speculated that the stability of the miRNAs may be confounding due to slightly different processing protocols in the two centres. However, this highlights even more the importance of standardized methodology. Another limitation of our study is the lack of a reliable housekeeping miRNA for normalization. Although some miRNAs are supposed to be stably expressed in human blood (e.g. miR-16), we could see significant variation of these miRNAs when measuring a large set of patient samples from our institution’s biobank (unpublished data). Therefore, we used cel-miR-39 as a spiked-in control as described above.

## Conclusion

This study aimed to investigate how variation in preparing and handling samples could influence the analysis of circulating miRNA in clinical practice. We found that miR-1, miR-21 and miR-29b levels are stable for at least 24 h at room temperature in whole blood. Storage of samples at -80°C extended the miRNA stability remarkably, however, miRNA levels in long-term stored (9 months) whole blood samples were significantly changed, which is in contrast to the plasma samples, where miRNA levels were found to be stable. Furthermore, we found that disturbance of the plasma/serum sample as well as repetitive freeze-thaw cycles result in reduced miRNA levels. This study highlights the importance of proper and standardized sample collection and preparation. Our data suggest that standardized procedures are required to overcome challenges with miRNA stability, thus enabling the use of miRNAs as non-invasive diagnostic, prognostic, and predictive biomarkers that provide valid results and the option of comparability.

### Future Implications

Based on our current study we suggest following a standardized protocol for blood collection, sample handling, processing and storage in order to obtain reproducible miRNA expression data:

1. Lithium heparin collection tubes should be avoided; for plasma collection we suggest to use either EDTA or citrate containing tubes;
2. Sample processing should be performed carefully. Contamination of plasma/serum samples with cellular fractions should be avoided;
3. Whole blood samples should not be stored at -80°C, but should be separated into plasma/serum fraction that can be stored at -80°C without quality loss for at least 9 months;
4. We recommend to process blood samples immediately into blood fractions and store them at -80°C. If immediate processing/freezing is not possible, whole blood samples can be kept at room temperature for up to 24 h. Separated plasma/serum samples, however, should be frozen within 24 h of separation;
5. Physical disturbance can affect the stability of miRNAs. We therefore recommend to minimize shaking of samples as far as possible. If transfer to another center is necessary, whole blood samples seem to be more stable than separated plasma/serum samples;
6. We recommend to aliquot plasma/serum samples before storage at -80°C since repetitive freezing-thawing affects miRNA stability.

## Supporting Information

S1 Table Different blood tubes for miRNA analysis. MicroRNA was isolated and levels of miR-1, miR-21 and miR-29b from either; EDTA-plasma, citrate-plasma, lithium-heparin-plasma, or serum fraction, were detected by RT-qPCR. Values were normalized to a spike-in control, cel-miR-39. Absolute C_T_ values for each are shown, demonstrating amplification from all sources, except lithium-heparin-plasma. Note: measurements for miR-21 and miR-1 in the Munich cohort were performed on the same participants but blood was collected at different days which made an additional cel-miR-39 measurement necessary. Measurement of miR-1 in serum failed in 2 participants. Li-Hep = lithium-heparin, CIT = citrate, n.a. = not available, - = not performed.
(DOCX)

S2 Table Stability of miRNA in whole blood incubated at room temperature. Whole blood was collected into EDTA containing tubes and incubated for 0, 4, 8, 12, 24 and 72 hours at room temperature before processed into plasma. qRT-PCR was performed. Absolute C_T_ values for each miRNAs are shown.
(DOCX)

S3 Table Impact of delayed processing (miR-21). Samples were processed immediately after blood collection and separated fractions were incubated for 24 h or 4 days (d) before RNA was isolated. Note: measurements for miR-21 and miR-1 in the Munich cohort were performed on the same participants but blood was collected at different days which made an additional cel-miR-39 measurement necessary. Measurements in EDTA whole blood, serum and serum whole blood failed in 2 participants.
(DOCX)

S4 Table Impact of delayed processing (miR-1). Samples were processed immediately after blood collection and separated fractions were incubated for 24 h or 4 days (d) before RNA was isolated. Note: measurements for miR-21 and miR-1 in the Munich cohort were performed on the same participants but blood was collected at different days which made an additional cel-miR-39 measurement necessary. Measurements in EDTA whole blood, serum and serum whole blood failed in 2 participants.
(DOCX)

S5 Table Impact of disturbance (miR-21). Blood samples were placed on a PMR 30 Mini Rocker Shaker (Grant Instruments, UK). After 1 and 8 h of shaking at 30 rpm RNA was isolated. Note: measurements for miR-21 and miR-1 in the Munich cohort were performed on the same participants but blood was collected at different days which made an additional cel-miR-39 measurement necessary. Measurements in EDTA whole blood (miR-21 and miR-1), serum and serum whole blood (only miR-1) failed in 2 participants.
(DOCX)

S6 Table Impact of disturbance (miR-1). Blood samples were placed on a PMR 30 Mini Rocker Shaker (Grant Instruments, UK). After 1 and 8 h of shaking at 30 rpm RNA was isolated. Note: measurements for miR-21 and miR-1 in the Munich cohort were performed on the same participants but blood was collected at different days which made an additional cel-miR-39 measurement necessary. Measurements in EDTA whole blood (miR-21 and miR-1), serum and serum whole blood (only miR-1) failed in 2 participants.
(DOCX)

S7 Table Incubation of plasma at -80°C for up to 9 months. Absolute C_T_ values for each microRNAs are shown.
(DOCX)

S8 Table Impact of repetitive freeze-thaw cycles. Blood samples were processed immediately after blood collection and aliquots of plasma and serum were stored at -80°C. One aliquot was thawed once, another aliquot was thawed and frozen four times before RNA was isolated. Note: measurements for miR-21 and miR-1 in the Munich cohort were performed on the same participants but blood was collected at different days which made an additional cel-miR-39 measurement necessary. Measurement of miR-1 in serum failed in 2 participants.
(DOCX)

S9 Table MiRNA levels in the 3 blood fractions; plasma, buffy coat and red blood cells (RBC). Absolute C_T_ values for each microRNA are shown.
(DOCX)
