# Supplementary material for: Stability of Circulating Blood-Based MicroRNAs – Pre-Analytic Methodological Considerations
Source: PLoS One. 2017 Feb 2;12(2):e0167969. doi: 10.1371/journal.pone.0167969 (PMC5289450; doi:10.1371/journal.pone.0167969)
Supplement: S1 Table — MicroRNA was isolated and levels of miR-1, miR-21 and miR-29b from either; EDTA-plasma, citrate-plasma, lithium-heparin-plasma, or serum fraction, were detected by RT-qPCR. Values were normalized to a spike-in control, cel-miR-39. Absolute CT values for each are shown, demonstrating amplification from all sources, except lithium-heparin-plasma. Note: measurements for miR-21 and miR-1 in the Munich cohort were performed on the same participants but blood was collected at different days which made an additional cel-miR-39 measurement necessary. Measurement of miR-1 in serum failed in 2 participants. Li-Hep = lithium-heparin, CIT = citrate, n.a. = not available, - = not performed. (DOCX) [file pone.0167969.s001.docx]

**S1 Table. Different blood tubes for miRNA analysis.**

|  | **cel-miR-39 (for miR-21/29b measurement)** | | | | **cel-miR-39 (for miR-1)** | | | | **hsa-miR-21** | | | | **hsa-miR-29b** | | | | **hsa-miR-1** | | | | |  |
| --- | --- | --- | --- | --- | --- | --- | --- | --- | --- | --- | --- | --- | --- | --- | --- | --- | --- | --- | --- | --- | --- | --- |
|  | **EDTA** | **CIT** | **LI-HEP** | **SERUM** | **EDTA** | **CIT** | **LI-HEP** | **SERUM** | **EDTA** | **CIT** | **LI-HEP** | **SERUM** | **EDTA** | **CIT** | **LI-HEP** | **SERUM** | | **EDTA** | **CIT** | **LI-HEP** | **SERUM** | |
| **1** | 24.09 | 23.90 | n.a. | 23.80 | - | - | - | - | 23.08 | 24.10 | n.a. | 23.11 | 32.17 | 33.53 | n.a. | 32.28 | | - | - | - | - | |
| **2** | 27.46 | 28,49 | n.a. | 27.95 | - | - | - | - | 24.70 | 28.57 | n.a. | 27.97 | 32.35 | 36.17 | n.a. | 34.76 | | - | - | - | - | |
| **3** | 25.10 | 24.57 | n.a. | 24.29 | - | - | - | - | 25.05 | 25.53 | n.a. | 25.15 | 34.16 | 33.91 | n.a. | 34.67 | | - | - | - | - | |
| **4** | 18.01 | - | n.a. | 25.74 | 18.45 | - | n.a. | 17.11 | 23.49 | - | n.a. | 29.19 | - | - | - | - | | 34.77 | - | n.a. | 34.39 | |
| **5** | 19.41 | - | n.a. | 21.12 | 19.39 | - | n.a. | 20.82 | 24.58 | - | n.a. | 27.36 | - | - | - | - | | 36.49 | - | n.a. | 33.68 | |
| **6** | 18.96 | - | n.a. | 28.69 | 19.09 | - | n.a. | 19.24 | 24.94 | - | n.a. | 35.59 | - | - | - | - | | 34.06 | - | n.a. | 36.31 | |
| **7** | 20.63 | - | n.a. | 27.74 | 20.74 | - | n.a. | 19.49 | 26.90 | - | n.a. | 30.02 | - | - | - | - | | 37.63 | - | n.a. | 35.15 | |
| **8** | 19.49 | - | n.a. | 30.44 | 19.48 | - | n.a. | n.a. | 25.10 | - | n.a. | 35.18 | - | - | - | - | | 35.06 | - | n.a. | n.a. | |
| **9** | 19.30 | - | n.a. | 31.01 | 19.48 | - | n.a. | n.a. | 24.33 | - | n.a. | 34.12 | - | - | - | - | | 32.01 | - | n.a. | n.a. | |

MicroRNA was isolated and levels of miR-1, miR-21 and miR-29b from either; EDTA-plasma, citrate-plasma, lithium-heparin-plasma, or serum fraction, were detected by RT-qPCR. Values were normalized to a spike-in control, cel-miR-39. Absolute C_T_ values for each are shown, demonstrating amplification from all sources, except lithium-heparin-plasma. Note: measurements for miR-21 and miR-1 in the Munich cohort were performed on the same participants but blood was collected at different days which made an additional cel-miR-39 measurement necessary. Measurement of miR-1 in serum failed in 2 participants. Li-Hep=lithium-heparin, CIT=citrate, n.a.=not available, -=not performed.
