# Supplementary material for: Stability of Circulating Blood-Based MicroRNAs – Pre-Analytic Methodological Considerations
Source: PLoS One. 2017 Feb 2;12(2):e0167969. doi: 10.1371/journal.pone.0167969 (PMC5289450; doi:10.1371/journal.pone.0167969)
Supplement: S2 Table — Whole blood was collected into EDTA containing tubes and incubated for 0, 4, 8, 12, 24 and 72 hours at room temperature before processed into plasma. qRT-PCR was performed. Absolute CT values for each miRNAs are shown. (DOCX) [file pone.0167969.s002.docx]

**S2 Table.** **Stability of miRNA in whole blood incubated at room temperature.**

|  | **cel-miR-39** | | | | | | **hsa-miR-21** | | | | | | **hsa-miR-29b** | | | | | |
| --- | --- | --- | --- | --- | --- | --- | --- | --- | --- | --- | --- | --- | --- | --- | --- | --- | --- | --- |
|  | **T0** | **T4** | **T8** | **T12** | **T24** | **T72** | **T0** | **T4** | **T8** | **T12** | **T24** | **T72** | **T0** | **T4** | **T8** | **T12** | **T24** | **T72** |
| **1** | 19.42 | 22.86 | 21.74 | 23.03 | 20.98 | 19.64 | 24.89 | 24.49 | 24.16 | 24.19 | 22.87 | 20.05 | 30.92 | 30.90 | 30.60 | 31.19 | 29.17 | 27.25 |
| **2** | 24.37 | 13.02 | 24.00 | 21.21 | 22.15 | 19.06 | 24.84 | 24.58 | 24.41 | 23.26 | 24.20 | 20.20 | 33.97 | 31.89 | 31.28 | 29.72 | 30.49 | 25.27 |
| **3** | 23.47 | 23.16 | 22.90 | 23.30 | 22.53 | 21.81 | 24.65 | 23.17 | 22.52 | 23.56 | 23.08 | 22.14 | 32.17 | 30.55 | 30.33 | 31.12 | 30.59 | 30.24 |
| **4** | 21.10 | 21.77 | 21.14 | 21.56 | 21.18 | 22.33 | 23.72 | 24.13 | 23.18 | 23.67 | 23.74 | 23.32 | 32.88 | 32.53 | 32.14 | 33.09 | 31.63 | 31.06 |
| **5** | 21.97 | 28.19 | 22.38 | 22.35 | 21.42 | 21.43 | 22.76 | 22.12 | 22.41 | 22.86 | 23.43 | 21.41 | 31.21 | 30.99 | 30.96 | 31.37 | 31.17 | 29.76 |
| **6** | 21.52 | 22.79 | 22.85 | 21.92 | 21.01 | 23.14 | 24.37 | 25.16 | 23.93 | 23.56 | 23.29 | 22.02 | 33.54 | 33.83 | 32.69 | 32.14 | 32.06 | 29.63 |

Whole blood was collected into EDTA containing tubes and incubated for 0, 4, 8, 12, 24 and 72 hours at room temperature before processed into plasma. qRT-PCR was performed. Absolute C_T_ values for each miRNAs are shown.
