# Supplementary material for: Stability of Circulating Blood-Based MicroRNAs – Pre-Analytic Methodological Considerations
Source: PLoS One. 2017 Feb 2;12(2):e0167969. doi: 10.1371/journal.pone.0167969 (PMC5289450; doi:10.1371/journal.pone.0167969)
Supplement: S3 Table — Samples were processed immediately after blood collection and separated fractions were incubated for 24 h or 4 days (d) before RNA was isolated. Note: measurements for miR-21 and miR-1 in the Munich cohort were performed on the same participants but blood was collected at different days which made an additional cel-miR-39 measurement necessary. Measurements in EDTA whole blood, serum and serum whole blood failed in 2 participants. (DOCX) [file pone.0167969.s003.docx]

**S3 Table.** **Impact of delayed processing (miR-21).**

|  |  | **miR-21** | | | | **cel-miR-39 (for measurement of miR-21)** | | | |
| --- | --- | --- | --- | --- | --- | --- | --- | --- | --- |
| **Group** | **proband** | **EDTA** | **EDTA whole blood** | **Serum** | **Serum whole blood** | **EDTA** | **EDTA whole blood** | **Serum** | **Serum whole blood** |
| **T0** | 1 | 23.49 |  | 29.19 |  | 18.01 |  | 25.74 |  |
|  | 2 | 24.58 |  | 27.36 |  | 19.41 |  | 21.12 |  |
|  | 3 | 24.94 |  | 35.59 |  | 18.96 |  | 28.69 |  |
|  | 4 | 26.90 |  | 30.02 |  | 20.63 |  | 27.74 |  |
|  | 5 | 25.10 |  | 35.18 |  | 19.49 |  | 30.44 |  |
|  | 6 | 24.33 |  | 34.12 |  | 19.30 |  | 31.01 |  |
| **24h** | 1 | 24.96 | 30.34 | 37.86 | 32.17 | 18.84 | 24.65 | 29.37 | 27.13 |
|  | 2 | 26.51 | 28.42 | 35.86 | 38.41 | 19.20 | 23.15 | 27.20 | 29.39 |
|  | 3 | 26.35 | 25.33 | 29.02 | 35.04 | 18.25 | 18.79 | 20.13 | 27.20 |
|  | 4 | 25.60 | 23.28 | 35.16 | 29.59 | 17.99 | 18.41 | 28.47 | 26.85 |
|  | 5 | 29.30 | n.a. | 39.57 | 32.16 | 18.67 | n.a. | 29.91 | 29.36 |
|  | 6 | 25.26 | n.a. | 33.96 | 33.51 | 19.15 | n.a. | 28.53 | 27.06 |
| **4d** | 1 | 25.66 | 30.43 | 35.26 | 36.77 | 18.40 | 23.62 | 24.28 | 27.01 |
|  | 2 | 25.89 | 30.08 | 38.45 | 37.68 | 17.42 | 23.51 | 26.52 | 27.45 |
|  | 3 | 31.59 | 23.83 | 34.87 | 38.16 | 23.86 | 17.43 | 27.76 | 30.02 |
|  | 4 | 24.46 | 26.97 | 39.08 | 35.69 | 17.89 | 19.40 | 27.30 | 26.26 |
|  | 5 | 32.90 | n.a. | 36.85 | 32.59 | 20.73 | n.a. | 25.02 | 25.59 |
|  | 6 | 24.36 | n.a. | 36.60 | 33.62 | 18.17 | n.a. | 27.92 | 27.26 |

Samples were processed immediately after blood collection and separated fractions were incubated for 24 h or 4 days (d) before RNA was isolated. Note: measurements for miR-21 and miR-1 in the Munich cohort were performed on the same participants but blood was collected at different days which made an additional cel-miR-39 measurement necessary. Measurements in EDTA whole blood, serum and serum whole blood failed in 2 participants.
