# Supplementary material for: Stability of Circulating Blood-Based MicroRNAs – Pre-Analytic Methodological Considerations
Source: PLoS One. 2017 Feb 2;12(2):e0167969. doi: 10.1371/journal.pone.0167969 (PMC5289450; doi:10.1371/journal.pone.0167969)
Supplement: S4 Table — Samples were processed immediately after blood collection and separated fractions were incubated for 24 h or 4 days (d) before RNA was isolated. Note: measurements for miR-21 and miR-1 in the Munich cohort were performed on the same participants but blood was collected at different days which made an additional cel-miR-39 measurement necessary. Measurements in EDTA whole blood, serum and serum whole blood failed in 2 participants. (DOCX) [file pone.0167969.s004.docx]

**S4 Table.** **Impact of delayed processing (miR-1).**

|  |  | **miR-1** | | | | **cel-miR-39 (for measurement of miR-1)** | | | |
| --- | --- | --- | --- | --- | --- | --- | --- | --- | --- |
| **Group** | **proband** | **EDTA** | **EDTA whole blood** | **Serum** | **Serum whole blood** | **EDTA** | **EDTA whole blood** | **Serum** | **Serum whole blood** |
| **T0** | 1 | 34.77 |  | 34.39 |  | 18.45 |  | 17.11 |  |
|  | 2 | 36.49 |  | 33.68 |  | 19.39 |  | 20.82 |  |
|  | 3 | 34.06 |  | 36.31 |  | 19.09 |  | 19.24 |  |
|  | 4 | 37.63 |  | 35.15 |  | 20.74 |  | 19.49 |  |
|  | 5 | 35.06 |  | n.a. | n.a. | 19.48 |  | n.a. | n.a. |
|  | 6 | 32.01 |  | n.a. | n.a. | 19.48 |  | n.a. | n.a. |
| **24h** | 1 | 38.35 | 39.82 | 37.84 | 36.09 | 18.82 | 18.60 | 18.42 | 18.52 |
|  | 2 | 39.58 | 37.77 | 37.16 | 35.63 | 19.22 | 17.96 | 18.90 | 18.23 |
|  | 3 | 38.98 | 34.71 | 36.08 | 35.17 | 18.23 | 18.41 | 18.46 | 18.02 |
|  | 4 | 37.37 | 32.74 | 38.01 | 36.82 | 18.01 | 19.31 | 20.14 | 20.48 |
|  | 5 | 35.09 | n.a. | n.a. | n.a. | 18.67 | n.a. | n.a. | n.a. |
|  | 6 | 35.08 | n.a. | n.a. | n.a. | 19.13 | n.a. | n.a. | n.a. |
| **4d** | 1 | 37.97 | 36.05 | 39.92 | 36.72 | 18.37 | 17.43 | 18.88 | 17.52 |
|  | 2 | 36.58 | 36.31 | 38.75 | 36.51 | 17.44 | 17.00 | 18.76 | 17.82 |
|  | 3 | 43.27 | 37.15 | 37.91 | 35.79 | 23.83 | 19.41 | 19.30 | 18.30 |
|  | 4 | 35.50 | 35.88 | 38.74 | 36.91 | 17.84 | 19.25 | 20.30 | 19.78 |
|  | 5 | 36.75 | n.a. | n.a. | n.a. | 20.68 | n.a. | n.a. | n.a. |
|  | 6 | 33.87 | n.a. | n.a. | n.a. | 18.15 | n.a. | n.a. | n.a. |

Samples were processed immediately after blood collection and separated fractions were incubated for 24 h or 4 days (d) before RNA was isolated. Note: measurements for miR-21 and miR-1 in the Munich cohort were performed on the same participants but blood was collected at different days which made an additional cel-miR-39 measurement necessary. Measurements in EDTA whole blood, serum and serum whole blood failed in 2 participants.
