# Supplementary material for: Stability of Circulating Blood-Based MicroRNAs – Pre-Analytic Methodological Considerations
Source: PLoS One. 2017 Feb 2;12(2):e0167969. doi: 10.1371/journal.pone.0167969 (PMC5289450; doi:10.1371/journal.pone.0167969)
Supplement: S5 Table — Blood samples were placed on a PMR 30 Mini Rocker Shaker (Grant Instruments, UK). After 1 and 8 h of shaking at 30 rpm RNA was isolated. Note: measurements for miR-21 and miR-1 in the Munich cohort were performed on the same participants but blood was collected at different days which made an additional cel-miR-39 measurement necessary. Measurements in EDTA whole blood (miR-21 and miR-1), serum and serum whole blood (only miR-1) failed in 2 participants. (DOCX) [file pone.0167969.s005.docx]

**S5 Table.** **Impact of disturbance (miR-21).**

|  |  | **miR-21** | | | | **cel-miR-39 (for measurement of miR-21)** | | | |
| --- | --- | --- | --- | --- | --- | --- | --- | --- | --- |
| **Group** | **proband** | **EDTA** | **EDTA whole blood** | **Serum** | **Serum whole blood** | **EDTA** | **EDTA whole blood** | **Serum** | **Serum whole blood** |
| **T0** | 1 | 23.49 |  | 29.19 |  | 18.01 |  | 25.74 |  |
|  | 2 | 24.58 |  | 27.36 |  | 19.41 |  | 21.12 |  |
|  | 3 | 24.94 |  | 35.59 |  | 18.96 |  | 28.69 |  |
|  | 4 | 26.90 |  | 30.02 |  | 20.63 |  | 27.74 |  |
|  | 5 | 25.10 |  | 35.18 |  | 19.49 |  | 30.44 |  |
|  | 6 | 24.33 |  | 34.12 |  | 19.30 |  | 31.01 |  |
| **24h** | 1 | 23.72 | 24.54 | 29.16 | 33.50 | 17.68 | 18.19 | 18.66 | 26.53 |
|  | 2 | 24.92 | 25.35 | 33.44 | 33.50 | 19.39 | 19.50 | 29.81 | 29.09 |
|  | 3 | 24.76 | 25.76 | 31.95 | 26.48 | 19.09 | 19.57 | 27.19 | 25.83 |
|  | 4 | 24.42 | 32.00 | 36.96 | 34.36 | 17.95 | 21.37 | 25.20 | 23.33 |
|  | 5 | 25.99 | 26.47 | 36.22 | 33.69 | 19.51 | 20.86 | 30.04 | 29.00 |
|  | 6 | 25.38 | 26.25 | 36.52 | 34.71 | 19.20 | 19.70 | 29.03 | 29.92 |
| **4d** | 1 | 24.33 | 22.15 | 37.01 | 34.14 | 18.64 | 18.00 | 31.31 | 28.11 |
|  | 2 | 26.99 | 26.24 | 36.48 | 37.92 | 19.39 | 20.99 | 29.27 | 29.16 |
|  | 3 | 26.29 | 31.40 | 34.91 | 35.12 | 19.51 | 22.69 | 27.34 | 26.66 |
|  | 4 | 26.32 | 25.55 | 28.25 | 34.32 | 19.21 | 20.18 | 19.48 | 28.43 |
|  | 5 | 31.85 | 30.44 | 35.80 | 34.32 | 23.21 | 23.25 | 23.74 | 28.43 |
|  | 6 | 25.85 | 27.19 | 37.35 | 34.31 | 18.48 | 22.45 | 28.29 | 28.33 |

Blood samples were placed on a PMR 30 Mini Rocker Shaker (Grant Instruments, UK). After 1 and 8 h of shaking at 30 rpm RNA was isolated. Note: measurements for miR-21 and miR-1 in the Munich cohort were performed on the same participants but blood was collected at different days which made an additional cel-miR-39 measurement necessary. Measurements in EDTA whole blood (miR-21 and miR-1), serum and serum whole blood (only miR-1) failed in 2 participants.
