# Supplementary material for: Stability of Circulating Blood-Based MicroRNAs – Pre-Analytic Methodological Considerations
Source: PLoS One. 2017 Feb 2;12(2):e0167969. doi: 10.1371/journal.pone.0167969 (PMC5289450; doi:10.1371/journal.pone.0167969)
Supplement: S6 Table — Blood samples were placed on a PMR 30 Mini Rocker Shaker (Grant Instruments, UK). After 1 and 8 h of shaking at 30 rpm RNA was isolated. Note: measurements for miR-21 and miR-1 in the Munich cohort were performed on the same participants but blood was collected at different days which made an additional cel-miR-39 measurement necessary. Measurements in EDTA whole blood (miR-21 and miR-1), serum and serum whole blood (only miR-1) failed in 2 participants. (DOCX) [file pone.0167969.s006.docx]

**S6 Table.** **Impact of disturbance (miR-1).**

|  |  | **miR-1** | | | | **cel-miR-39 (for measurement of miR-1)** | | | |
| --- | --- | --- | --- | --- | --- | --- | --- | --- | --- |
| **Group** | **proband** | **EDTA** | **EDTA whole blood** | **Serum** | **Serum whole blood** | **EDTA** | **EDTA whole blood** | **Serum** | **Serum whole blood** |
| **T0** | 1 | 34.77 |  | 34.39 |  | 18.45 |  | 17.11 |  |
|  | 2 | 36.49 |  | 33.68 |  | 19.39 |  | 20.82 |  |
|  | 3 | 34.06 |  | 36.31 |  | 19.09 |  | 19.24 |  |
|  | 4 | 37.63 |  | 35.15 |  | 20.74 |  | 19.49 |  |
|  | 5 | 35.06 |  | n.a. | n.a. | 19.48 |  | n.a. | n.a. |
|  | 6 | 32.01 |  | n.a. | n.a. | 19.48 |  | n.a. | n.a. |
| **24h** | 1 | 34.58 | 35.33 | 33.44 | 34.00 | 17.70 | 18.20 | 17.42 | 17.72 |
|  | 2 | 36.91 | 37.02 | 33.52 | 35.15 | 19.36 | 19.48 | 19.29 | 20.98 |
|  | 3 | 34.48 | 35.06 | 36.79 | 34.77 | 19.10 | 19.52 | 19.22 | 19.39 |
|  | 4 | 34.20 | 39.82 | 36.14 | 34.60 | 17.93 | 21.17 | 20.30 | 20.43 |
|  | 5 | 36.81 | 36.94 | n.a. | n.a. | 19.40 | 20.63 | n.a. | n.a. |
|  | 6 | 32.46 | 32.91 | n.a. | n.a. | 19.18 | 19.69 | n.a. | n.a. |
| **4d** | 1 | 37.81 | 35.19 | 42.48 | 35.97 | 18.66 | 18.02 | 18.30 | 17.86 |
|  | 2 | 41.56 | 38.43 | 33.58 | 36.88 | 19.43 | 21.06 | 19.20 | 19.91 |
|  | 3 | 38.26 | 36.58 | 38.51 | 36.64 | 19.53 | 22.72 | 20.16 | 18.69 |
|  | 4 | 35.31 | 37.53 | 35.28 | 36.56 | 19.29 | 20.15 | 19.74 | 19.61 |
|  | 5 | 41.85 | 38.16 | n.a. | n.a. | 23.18 | 23.24 | n.a. | n.a. |
|  | 6 | 36.47 | 40.38 | n.a. | n.a. | 18.56 | 22.45 | n.a. | n.a. |

Blood samples were placed on a PMR 30 Mini Rocker Shaker (Grant Instruments, UK). After 1 and 8 h of shaking at 30 rpm RNA was isolated. Note: measurements for miR-21 and miR-1 in the Munich cohort were performed on the same participants but blood was collected at different days which made an additional cel-miR-39 measurement necessary. Measurements in EDTA whole blood (miR-21 and miR-1), serum and serum whole blood (only miR-1) failed in 2 participants.
