# Supplementary material for: Stability of Circulating Blood-Based MicroRNAs – Pre-Analytic Methodological Considerations
Source: PLoS One. 2017 Feb 2;12(2):e0167969. doi: 10.1371/journal.pone.0167969 (PMC5289450; doi:10.1371/journal.pone.0167969)
Supplement: S7 Table — Absolute CT values for each microRNAs are shown. (DOCX) [file pone.0167969.s007.docx]

**S7 Table. Incubation of plasma at -80°C for up to 9 months.**

|  | **Mir-39** | | | | **Mir-21** | | | | | **Mir-29** | | | | |
| --- | --- | --- | --- | --- | --- | --- | --- | --- | --- | --- | --- | --- | --- | --- |
|  | **1 day EDTA** | **9 months EDTA** | **1 day whole blood** | **9 months whole blood** | | **1 day EDTA** | **9 months EDTA** | **1 day whole blood** | **9 months whole blood** | | **1 day EDTA** | **9 months EDTA** | **1 day whole blood** | **9 months whole blood** |
| **1** | 24.04 | 22.02 | 25.49 | 29.21 | | 27.03 | 23.86 | 21.59 | 21.17 | | 32.65 | 31.07 | 26.01 | 29.20 |
| **2** | 22.08 | 22.00 | 19.90 | 27.11 | | 26.11 | 24.62 | 19.78 | 20.99 | | n.a. | 32.02 | 27.50 | 28.49 |
| **3** | 23.05 | 21.38 | 30.48 | 29.42 | | 28.70 | 23.74 | 25.91 | 22.55 | | 33.65 | 30.07 | 32.33 | 29.20 |
| **4** | 23.39 | 20.65 | 28.80 | 34.51 | | 26.63 | 23.91 | 23.38 | 27.02 | | 33.53 | 32.08 | 30.15 | 33.74 |
| **5** | 24.10 | 21.15 | 29.94 | 26.75 | | 26.27 | 22.88 | 25.02 | 19.66 | | 32.94 | 29.24 | 31.46 | 27.11 |
| **6** | 22.84 | 20.40 | 26.09 | 26.71 | | 25.85 | 23.67 | 23.13 | 19.58 | | 32.21 | 30.00 | 29.34 | 26.66 |

Absolute C_T_ values for each microRNAs are shown.
