# Supplementary material for: Stability of Circulating Blood-Based MicroRNAs – Pre-Analytic Methodological Considerations
Source: PLoS One. 2017 Feb 2;12(2):e0167969. doi: 10.1371/journal.pone.0167969 (PMC5289450; doi:10.1371/journal.pone.0167969)
Supplement: S8 Table — Blood samples were processed immediately after blood collection and aliquots of plasma and serum were stored at -80°C. One aliquot was thawed once, another aliquot was thawed and frozen four times before RNA was isolated. Note: measurements for miR-21 and miR-1 in the Munich cohort were performed on the same participants but blood was collected at different days which made an additional cel-miR-39 measurement necessary. Measurement of miR-1 in serum failed in 2 participants. (DOCX) [file pone.0167969.s008.docx]

**S8 Table. Impact of repetitive freeze-thaw cycles**.

|  |  | **miR-21** | | **cel-miR-39 (for measurement of miR-21)** | | **miR-1** | | **cel-miR-39 (for measurement of miR-1)** | |
| --- | --- | --- | --- | --- | --- | --- | --- | --- | --- |
| **Group** | **proband** | **EDTA** | **Serum** | **EDTA** | **Serum** | **EDTA** | **Serum** | **EDTA** | **Serum** |
| **T0** | 1 | 23.49 | 29.19 | 18.01 | 25.74 | 34.77 | 34.39 | 18.45 | 17.11 |
|  | 2 | 24.58 | 27.36 | 19.41 | 21.12 | 36.49 | 33.68 | 19.39 | 20.82 |
|  | 3 | 24.94 | 35.59 | 18.96 | 28.69 | 34.06 | 36.31 | 19.09 | 19.24 |
|  | 4 | 26.90 | 30.02 | 20.63 | 27.74 | 37.63 | 35.15 | 20.74 | 19.49 |
|  | 5 | 25.10 | 35.18 | 19.49 | 30.44 | 35.06 | n.a. | 19.48 | n.a. |
|  | 6 | 24.33 | 34.12 | 19.30 | 31.01 | 32.01 | n.a. | 19.48 | n.a. |
| **24h** | 1 | 25.35 | 35.67 | 18.18 | 28.40 | 35.47 | 34.32 | 18.14 | 16.88 |
|  | 2 | 24.79 | 34.43 | 18.63 | 21.63 | 36.35 | 33.64 | 18.60 | 19.51 |
|  | 3 | 24.26 | 34.94 | 17.79 | 27.81 | 34.19 | 36.64 | 17.76 | 19.74 |
|  | 4 | 26.68 | 36.87 | 20.08 | 27.45 | 36.86 | 36.35 | 20.05 | 20.37 |
|  | 5 | 23.78 | 37.52 | 18.20 | 29.73 | 34.50 | n.a. | 18.48 | n.a. |
|  | 6 | 25.02 | 36.24 | 18.70 | 28.19 | 32.46 | n.a. | 18.97 | n.a. |

Blood samples were processed immediately after blood collection and aliquots of plasma and serum were stored at -80°C. One aliquot was thawed once, another aliquot was thawed and frozen four times before RNA was isolated. Note: measurements for miR-21 and miR-1 in the Munich cohort were performed on the same participants but blood was collected at different days which made an additional cel-miR-39 measurement necessary. Measurement of miR-1 in serum failed in 2 participants.
