# Supplementary material for: Stability of Circulating Blood-Based MicroRNAs – Pre-Analytic Methodological Considerations
Source: PLoS One. 2017 Feb 2;12(2):e0167969. doi: 10.1371/journal.pone.0167969 (PMC5289450; doi:10.1371/journal.pone.0167969)
Supplement: S9 Table — Absolute CT values for each microRNA are shown. (DOCX) [file pone.0167969.s009.docx]

**S9 Table. MiRNA levels in the 3 blood fractions; plasma, buffy coat and red blood cells (RBC).**

|  | **miR-39** | | | **miR-21** | | | **miR-29** | | |
| --- | --- | --- | --- | --- | --- | --- | --- | --- | --- |
|  | **Plasma** | **Buffycoat** | **RBC** | **Plasma** | **Buffycoat** | **RBC** | **Plasma** | **Buffycoat** | **RBC** |
| **1** | 24.69 | 27.30 | 36.81 | 20.05 | 18.05 | 27.53 | 29.05 | 23.62 | 36.67 |
| **2** | 23.30 | 30.33 | 34.75 | 19.04 | 21.43 | 24.31 | 24.87 | 25.20 | 30.07 |
| **3** | 23.27 | 25.00 | 35.60 | 19.01 | 17.49 | 26.00 | 25.44 | 22.39 | 35.40 |

Absolute C_T_ values for each microRNA are shown.
